# The association of clinical frailty with outcomes of patients reviewed by rapid response teams: an international prospective observational cohort study

**DOI:** 10.1186/s13054-018-2136-4

**Published:** 2018-09-22

**Authors:** Ralph K. L. So, Jonathan Bannard-Smith, Chris P. Subbe, Daryl A. Jones, Joost van Rosmalen, Geoffrey K. Lighthall, the METHOD study investigators, M. Trivedi, H. Ponssen, R. So, K. Koster, S. Smit, S. Michail, S. Tunstall, E. Davies, J. Bannard-Smith, C. Rowley, M. Verheijen, C. Chalmers, A. Elguea, R. Ulka, A. R. Silva, T. van Zuylen, P. de Jager, J. Lennon, A. de Gooijer, C. Plowright, C. Battle, D. Sep, A. M. Kodal, V. Jones, K. Kemsley, R. Verhage, E. Brull, T. van Zon, P. Tangkau, M. Wilson, J. Burke, A. Hurding, A. Jordan, K. Kemsley, J. van Vliet, S. Iverson, J. Philips, R. DuncanM Donnelly, K. Edwards, D. J. Mehagnoul, P. Hart, L. Collins, H. Reddy, E. Young, C. Subbe

**Affiliations:** 1 Department of Intensive Care, Albert Schweitzer Hospital, Albert Schweitzerplaats 25, Dordrecht, the Netherlands; 2 Department of Critical Care, Manchester Royal Infirmary, Central Manchester University NHS Hospitals, Manchester Academic Health Science Centre, Manchester, M13 9WL UK; 3 Acute, Respiratory & Critical Care Medicine, Ysbyty Gwynedd, Bangor, UK; 4 Department of Intensive Care, Austin Hospital, 145 Studley Rd, Heidelberg, VIC Australia; 5 Department of Biostatistics, Erasmus Medical Centre, Rotterdam, the Netherlands; 6 Department of Anesthesia, Stanford University School of Medicine, 300 Pasteur Dr. H3580, Stanford, CA 94305 USA

**Keywords:** Frailty, Rapid response team, Acute illness, Advanced directives, Outreach team, Medical emergency team

## Abstract

**Background:**

Frailty is a state of vulnerability to poor resolution of homeostasis after a stressor event and is strongly associated with adverse outcomes. Therefore, the assessment of frailty may be an essential part of evaluation in any healthcare encounter that might result in an escalation of care. The purpose of the study was to assess the frequency and association of frailty with clinical outcomes in patients subject to rapid response team (RRT) review.

**Methods:**

In this multi-national prospective observational cohort study, centres with existing RRTs collected data over a 7-day period, with follow up of all patients at 24 h following their RRT call and at hospital discharge or 30 days following the event trigger (whichever came sooner). Investigators also collected data on the triggers and interventions provided and a bedside assessment on the level of patients’ frailty using a clinical frailty scale.

**Results:**

Amongst 1133 patients, 40% were screened as frail, which was associated with older age (*p* < 0.001), admission under a medical speciality (*p* < 0.001), increased severity of illness at the time of the RRT review (*p* = 0.0047), and substantially higher frequency of limitations of care (*p* < 0.001). Importantly, 72% of patients screened as frail were either dead or dependent on hospital care by 30 days (*p* < 0.001). In the multivariable analysis, the significant risk factors for the composite endpoint “poor recovery” (died or were hospital-dependent by 30 days) were age (odds ratio (OR), 1.04; 95% confidence interval (CI), 1.03–1.05; *p* < 0.001), frailty level (*p* < 0.001), existing limitation of care (OR, 2.0; 95% CI, 1.3–3.0; *p* < 0.001), and the quick sequential organ failure assessment (qSOFA) score (*p* < 0.001).

**Conclusions:**

Higher frailty scores were associated with increased mortality and dependence on health care at 30 days. Our results indicate that frailty has an influence on the clinical trajectory of deteriorating patients and that such assessment should be included in discussion of goals and expectations of care.

**Trial registration:**

Netherlands Trial Registry, NTR5535. Registered on 23 December 2015.

## Background

Hospitals manage patients with increasingly complex medical needs. Some of the increase in the overall patient acuity can be accounted for by the increase in ambulatory surgery and the substitution of outpatient for inpatient care and some by the outpatient management in medical cases of less seriously ill patients who would previously would have been hospitalised. In this environment recognition of clinically important deterioration is becoming more challenging, diagnostic processes more complex and full recovery to good health more difficult to achieve. Many hospitals worldwide have introduced rapid response teams (RRTs) to identify and respond to patients who are experiencing important clinical deterioration, particularly on the hospital wards.

Response to treatment depends amongst other factors on timely intervention and the reversibility of a condition. Reversibility may be influenced by physiological reserve - an entity that is not directly measured - but which diminishes with age and significant comorbidities, and manifests as clinical frailty. Frailty can be measured as the sum of acquired functional deficits and is related to mortality after hospital or intensive care admission and the need for support at home [1–3]. Although the impact of frailty has been assessed in the hospital setting, the impact of frailty has not been described in the context of rapid response systems.

METHOD is an international service evaluation that records and compares outcomes of patients reviewed by RRTs. In a 2014 study, sites collected data during a 7-day period with follow up at 24 h after each RRT review. Results described 1188 RRT activations from 51 hospitals in 5 countries; 24% of patients were admitted to the ICU, 10% died, and 25% had new limitations in therapy implemented [4]. A limitation of the METHOD 2014 study was the lack of data on longer-term outcomes, case-mix adjustment, and consideration of the importance of clinical frailty.

The purpose of this study was to assess the epidemiology of frailty in patients subject to RRT review. Specifically, we assessed the frequency and distribution of frailty, before making a comparison of outcomes for patients judged to have high versus low levels of frailty. In addition, we investigated whether frailty was associated with “poor recovery” (death or becoming hospital-dependent) at 30 days, after adjustment for potentially confounding variables.

## Methods

### Study design, infrastructure, and coordination

In this international prospective observational cohort study, centres with existing RRTs were invited to collect data during a 7-day period in February–March 2016. Expressions of interest were initially obtained from sites that contributed to a previous study [4]. The study was also promoted on the websites of the International Society for Rapid Response Systems (http://rapidresponsesystems.org) and the UK National Outreach Forum (http://www.norf.org.uk).

All patients triggering RRT review at each site during the study period were included. RRTs followed up all patients at 24 h following their call and at hospital discharge or 30 days following the event trigger (whichever came sooner). RRTs at participating sites collected data using paper-based case report forms for each patient. Sites then anonymised and submitted all data via an encrypted electronic database for central analysis. The management and writing committee, consisting of all authors of the paper, oversaw the study. The committee directed study design, review and promulgation of the study protocol, collation of results, generation of data queries, resolution of data queries with study sites, data analysis, and writing of the manuscript.

### Nature of data collected

Each participating site provided information on the characteristics of their institution and the principal model of their RRT. Each patient was identified using a unique patient identifier, with the patient’s identity kept secure and only discoverable locally by the contributing centre. Individual sites kept a master list of subjects and could re-identify patients during the data query process if required. Data were collected on demographics including age, gender, source of admission, parent unit, and date of hospital admission. We recorded the date and time of the RRT call and the resuscitation status of the patient before the RRT call (that is, for full active care, for limited critical care, not for critical care, or do not attempt resuscitation).

Frailty can be defined by a frailty phenotype or accumulation of deficits model to calculate a frailty index [5]. The latter uses accumulation of 70 deficits including functional deficits and chronic diseases as a model of frailty. The clinical frailty scale (CFS) is a clinical derivative designed initially as a screening tool, and correlates highly with the frailty index. Frailty was measured using the CFS (see Appendix) based on information provided by either the patient or family members. This 9-point scale contains categories for severely ill patients added on to Rockwood’s original 7-point scale. The latter was evaluated prospectively in a large cohort, where each increment was associated with both higher mortality and a greater need for long-term institutional care at 70 months [6]. In critically ill patients, a cutoff point ≥ 5 has been associated with shorter-term survival including hospital mortality [1–3]. Given the shorter-term follow up of the present study design, and potential ambiguity in family members’ ability to identify early frailty, we therefore compared outcomes of patients attended by the RRT, who had a frailty level 1–4 versus those with levels ≥ 5. The lead authors of our study tested this frailty scale along with the other data collection documents on patients in their own institutions prior to the study opening. Lead investigators at each site were provided with written information and graphical illustrations of the CFS. Local investigators and members of their respective rapid response teams (RRTs) were responsible for conducting frailty assessments using information available at the time of RRT activation. This included documented evidence from the patient’s medical record including history from the patient and/or their relatives and assessments by nursing staff and allied health professionals such as physiotherapists and occupational therapists.

Vital signs, oxygen use, and mental status at the time of arrival of the RRT were recorded, and from these we calculated the UK National Early Warning Score (NEWS) [7] and abbreviated organ failure assessment score (qSOFA), as additional analysis parameters [8]. The time between the call to the RRT and subsequent transfer to ICU was calculated and compared in those patients that were admitted; the analysis used a time greater than 4 h as a cutoff point for “delayed transfer to the ICU” [9]. The analysis also considered the risks of weekend calls (5.00 p.m. Friday to 6.00 a.m. Monday) and night calls (midnight to 6.00 a.m.).

We also recorded whether the patient was transferred to an ICU or operating room in the following 24 h and the date and time of such events. If there was a perceived delay, we recorded this as either: “no availability of critical care bed/operating theatre”, or that the “patient was initially stable on the ward”, there was “requirement for an initial investigation”, or “requirement for an initial intervention”.

For patients not admitted to an intensive care unit, we verified whether the patient died within 24 h of the first call and whether the death occurred with a do not attempt resuscitation (DNAR) order in place, whether the initial call trigger resolved, whether new or increased limitations of medical therapy were instituted, and whether there was another RRT call within the next 24 h. For patients who died without a valid DNAR order, we recorded whether cardiopulmonary resuscitation was performed.

### Statistical analysis

Data from individual sites were compiled in a single record with the addition of a country and site code and patient serial numbers. All statistical analyses were performed using SPSS (v.24). The continuous variables are expressed as mean ± standard deviation (for normally distributed variables) or as median and IQR (for variables that are not normally distributed), and the categorical variables are expressed as numbers (percentages). The patients were divided into two independent groups, namely, frail and nonfrail patients, based on a CFS score of 1–4 or ≥ 5, respectively. For descriptive statistics, the categorical variables were compared between frailty groups using the chi-square test or Fisher‘s exact test, as appropriate. The continuous variables were compared between frailty groups using the independent samples Student *t* test (for normally distributed variables) or the Mann-Whitney U test (for variables that are not normally distributed).

Multivariable logistic regression analysis was performed to determine risk factors for the composite endpoint of "poor recovery" (patients who had died or become hospital-dependent) at 30 days; this analysis was also done for the outcomes of mortality at 24 h, mortality at 30 days, and hospital dependence at 30 days. The independent variables were UK as the country, age, male, admitted under a medical specialty, frailty level, patients per nurse, existing limitation of care, National Early Warning Score (NEWS), qSOFA score, weekend calls, and night calls. The model fit was assessed using the Hosmer-Lemeshow test. A two-sided *p* value <0.05 was considered to be statistically significant for all of the comparisons.

Analysis was carried out on the entire patient sample, and subsequently in those who survived 30 days. The latter group was evaluated for non-resolution of illness at the 30-day point; the end point “hospital dependence” was used to define those at that time that remained in the hospital, were transferred to another hospital, or who received skilled nursing or hospice care.

## Results

### Demographics

The study accrued data in 2016 from 1133 patients from 43 different medical institutions across 8 countries. Three nations contributed more than 100 patients with the overall distribution shown in Table 1A.

**Table 1:** Patients seen by rapid response teams: demographics and interventions

	Total	UK	Netherlands	Denmark	Australia	Other
A. Demographics						
Number of patients	1133	722	199	124	59	29
Number of centres	43	23	10	4	2	4
Age (years)	67 (18)	67 (19)	67 (15)	71(13)	64 (21)	62 (18)
Male	581 (51%)	364 (50%)	104 (52%)	70 (56%)	30 (51%)	13 (45%)
Originally from home	950 (84%)	608 (84%)	179 (90%)	89 (72%)	49(83%)	24 (83%)
Admitted under a medical specialty.	764 (67%)	500 (69%)	123 (62%)	92 (74%)	30 (51%)	19 (66%)
Frailty level, percent that were ≥ 5^a^	40%	41%	32%	50%	29%	41%
Patients per nurse, percent at 1–4/5–8/≥ 9	28/52/20 (%)	20/61/19 (%)	29/40/31 (%)	47/34/20 (%)	71/25/0 (%)	48/41/10 (%)
Existing care limitation in place	208 (18%)	122 (17%)	34 (17%)	35 (28%)	11 (19%)	6 (21%)
Mean NEWS (SD)	6.7 (3.2)	6.1 (3.1)	7.7 (3.0)	8.8 (302)	6.5 (3.3)	6.9 (2.8)
Mean qSOFA (SD)	1.2 (0.8)	1.1 (0.8)	1.3 (0.8)	1.3 (0.8)	1.2 (0.8)	1.2 (0.7)
On antibiotics prior to RRT call	648 (57%)	440 (61%)	108 (54%)	68 (55%)	18 (31%)	14 (55%)
B. Interventions						
On antibiotics following RRT call	659 (58%)	485 (67%)	119 (60%)	31 (25%)	8 (14%)	14 (48%)
Transferred to ICU	339 (30%)	151 (21%)	120 (60%)	42 (34%)	15 (25%)	11 (38%)
Mean RRT-ICU time (h)	9.2	12.5	3.4	6.3	24.8	13.1
Needed surgical operation	21 (2%)	10 (1%)	6 (3%)	3 (2%)	2 (3%)	0
Full code death	16 (1%)	12 (2%)	1 (1%)	2 (2%)	1 (2%)	0
Received CPR	15 (1%)	11 (2%)	2 (1%)	1 (1%)	1 (2%)	0
Call trigger persisted	253 (22%)	145 (20%)	24 (12%)	60 (48%)	17 (29%)	7 (24%)
New limitation in care	188 (17%)	136 (19%)	16 (8%)	23 (19%)	9 (15%)	4 (14%)
Repeat RRT call	95 (8%)	73 (10%)	9 (5%)	8 (6%)	3 (5%)	2 (7%)

Demographics of study patients are shown in total and according to nation. The final column is the sum of countries contributing less than 25 patients each to the analysis, and includes Mexico (16 patients), Ireland (7 patients), Portugal (5 patients), and the USA (1 patient)

*RRT* rapid response team, *qSOFA* quick sequential organ failure assessment, *CFS* clinical frailty scale, *NEWS* National Early Warning Score, *CPR* cardiopulmonary resuscitation

^a^Frailty scores were determined using the Dalhousie clinical frailty scale and condensed into two intervals as noted in “Methods”

### Inpatient characteristics

Patient characterististics are shown in Table 1. Of note, 67% of patients (764/1133) were admitted under medical specialties. Overall, 18% of patients (208/1133) had existing limitations of care prior to the RRT call. The mean NEWS score at the time of the RRT review was 6.7 ± 3.2 (SD).

### Outcomes at 24 h and care escalation

Cardiac arrests occurred in 1.4% of patients (16/1133) undergoing RRT review; all but 1 (15 out of 16) of these patients received cardiopulmonary resuscitation (CPR). Repeat calls occurred in 8% of all patients (95/1133); call triggers persisted at 24 h in 22% of instances (253/1133). Death occurred within 24 h in 72/1133 study patients (6.4%). After the RRT call, a new limitation in care was implemented in 17% of patients (188/1133). Overall, 30% of patients (339/1133) were transferred to the ICU within 24 h of RRT review (see Table 1B).

### Frequency and consequences of frailty

Data on levels of frailty were available for 99% of patients (1119/1133) and 60% (672/1119) were screened as non-frail with frailty scores of 1–4, and 40% (447/1119) were screened as frail with scores ≥ 5. Comparing these two groups, patients screened as frail were more likely to have a higher mean age (74 versus 63 years), admission under a medical specialty, existing limitations of care, higher qSOFA scores, new limitations in care, and poor recovery from illness (*p* < 0.001 for all; see Table 2). Patients screened as frail were also more likely to have a higher NEWS score (*p* = 0.0047; see Table 2), and higher nurse-to-patient ratio (23% of frail patients had a patients-per-nurse load of 1–4 vs. 30% of non-frail patients; *p* = 0.0048, see Table 2).

**Table 2:** Presence and impact of frailty in patients seen by rapid response teams

Variable	Frailty level 1–4	Frailty level ≥ 5	*p* value
Count	*n* = 672 (60%)	*n* = 447 (40%)	
Age (years)	63 (18)	74 (15)	*p* < 0.001
Male	263 (54%)	215 (48%)	*p* = 0.051
On a medical service	390 (58%)	335 (75%)	*p* < 0.001^b^
Patients per nurse			
1–4	203 (30%)	101 (23%)	*p* = 0.0048^b^
5–8	330 (50%)	248 (55%)	*p* = 0.031^b^
9–12	103 (15%)	69 (15%)	*p* = 1.0
13–17	27 (5%)	20 (5%)	*p* = 0.716
> 17	0	3	*P* = 0.060
Existing limitation in care	54 (8%)	156 (35%)	*p* < 0.001^b^
Resolution of trigger	296 (44%)	192 (43%)	*p* = 0.950
Repeat MET call	54 (8%)	40 (9%)	*p* = 0.440
Median NEWS (IQR)	6 (4–9)	7 (5–9)	*p* = 0.0047^a^
Mean qSOFA score (SD)	1.1 (.78)	1.4 (.82)	*p* < 0.001^a^
On antibiotics following MET call	383 (57%)	273 (61%)	*p* = 0.216
Antibiotics before MET call	370 (55%)	273 (61%)	*p* = 0.081
ICU admission	242 (36%)	94 (21%)	*p* < 0.001^b^
Mean MET to ICU interval (h)	10.0	6.7	*p* = 0.701
MET– > ICU less than 4 h.	181 (27%)	125 (28%)	*p* = 0.892
Died within 24 h of MET call	24 (4%)	48 (11%)	*p* < 0.001^b^
Full code status at time of death	5 (0.7%)	8 (1.8%)	*p* = 0.153
Received CPR	6 (0.9%)	9 (2.0%)	*p* = 0.119
New limitation in care	74 (11%)	116 (26%)	*p* < 0.001^b^
Died within 30 days	144 (21%)	177 (40%)	*p* < 0.001^b^
If alive, hospital-dependent at 30 days	139 (26%)	144 (32%)	*p* < 0.001^b^
Died or hospital-dependent at 30 days	283 (42%)	321 (72%)	*p* < 0.001^b^

Patients seen by rapid response teams were assessed by the Dalhousie clinical frailty scale and analysed according to scores of 1–4 and ≥ 5; 14 of the 1133 patients in this study had missing frailty data and 15 had missing data on the nurse-to-patient ratio

*MET* Medical emergency team, *qSOFA* quick sequential organ failure assessment, *NEWS* National Early Warning Score, *CPR* cardiopulmonary resuscitation

^a^Mann-Whitney U test

^b^Fisher’s exact test

ICU admissions were less common in patients in the higher frailty class (21% vs. 36%, *p* < 0.001). In addition, patients with a frailty score ≥ 5 were more likely to die within 24 h (11% vs. 4%, *p* < 0.001) and within 30 days of RRT review (40% vs. 21%, *p* < 0.001) compared to those patients with scores of 1–4.

### Associations with mortality and hospital dependence at 30 days

Of the 1133 study patients, 6% (72) died within 24 h, and 16% (12) of these deaths were unexpected (died with “full code” status). At 30 days, 29% of the patients (321) had died and 25% of the patients (283) were still in the hospital or dependent on skilled nursing (including hospice care; see Table 3).

**Table 3:** Association between frailty levels and clinical end points

CFS	Total	Died within 24 h	Died within 30 days	Hospital-dependent at 30 days	Poor recovery
1–2	312	6 (2%)	41(13%)	49 (16%)	90 (29%)
3–4	360	18 (5%)	103 (29%)	90 (25%)	193(54%)
5–6	287	29 (10%)	113 (39%)	80 (28%)	193 (67%)
7–9	160	19 (12%)	64 (40%)	64 (40%)	128 (80%)
	1119	72	321	283	604

Clinical end points evaluated are shown as well as their distribution amongst two-step intervals of the clinical frailty scale (CFS). “Poor recovery” is a composite endpoint indicating either hospital dependence or mortality at 30 days

In the multivariable logistic regression analysis, variables significantly associated with the composite endpoint of poor recovery (died or hospital-dependent at 30 days) were age (OR,1.04; 95% CI, 1.03–1.05; *p* < 0.001), existing limitation of care (OR, 2.0; 95% CI, 1.3–3,0; *p* < 0.001), and qSOFA score (*p* < 0.001). Compared to patients considered very fit and well by the frailty scale (levels 1 and 2), each two-step increase in frailty had a near doubling of risk of mortality or dependence on formal care services at 30 days (OR range 2.9–9.9; see Table 4).

**Table 4:** Univariable and multivariable logistic regression analysis for “poor recovery”

Variable	Univariable			Multivariable		
	OR	95% CI	*p* value	OR	95% CI	*p* value
UK country	0.8	0.6–1.0	0.057	0.8	0.6–1.1	0.151
Age	1.0	1.0–1.1	< 0.001	1.04	1.03–1.05	< 0.001
Male	1.1	0.8–1.4	0.548			
Medical admission	1.4	1.1–1.8	0.013			
Frailty level^a^						< 0.001
1–2	Reference			Reference		
3–4	2.9	2.1–3.9	< 0.001	1.8	1.2–2.5	< 0.001
5–6	5.1	3.6–7.2	< 0.001	2.2	1.5–3.4	< 0.001
7–9	9.9	6.2–16.6	< 0.001	5.1	3.1–8.6	< 0.001
Patients per nurse^b^						
1–4	Reference					
5–8	1.3	1.0–1.7	0.065			
9–12	1.3	0.9–1.9	0.142			
≥ 13	1.3	0.7–2.4	0.384			
Existing limitation of care	4.5	3.1–6.4	< 0.001	2.0	1.3–3.0	< 0.001
NEWS	1.1	1.0–1.1	< 0.001			
qSOFA score	1.8	1.5–2.1	< 0.001			< 0.001
1				1.7	1.1–2.4	0.009
2				2.4	1.6–3.7	< 0.001
3				4.3	2.1–9.1	< 0.001
Weekend calls	1.0	0.8–1.3	0.861			
Night calls	0.8	0.6–1.1	0.104	0.7	0.5–1.0	0.065

Univariable and multivariable analysis for “poor recovery”. The Hosmer-Lemeshow test suggested a good fit (*p* = 0.29)

*qSOFA* quick sequential organ failure assessment, *NEWS* National Early Warning Score

^a^Frailty levels were compared for the proportion with 1–2 versus higher levels

^b^Nursing ratios were compared for the proportion with 1–4 patients per nurse versus higher loads

## Discussion

### Summary of major findings

**Fig. 1:**
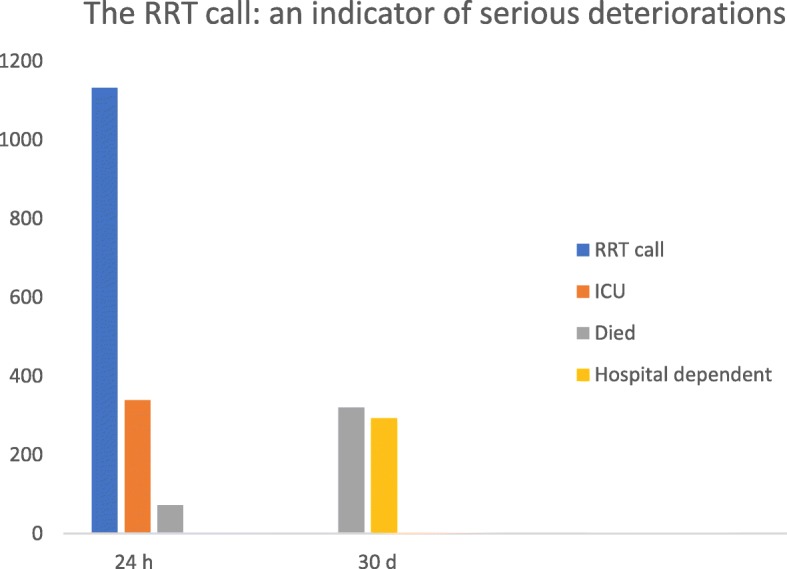
Key patient outcomes during the first 24-h of an emergency team call and 30-day outcomes are presented. RRT, rapid response team

**Fig. 2:**
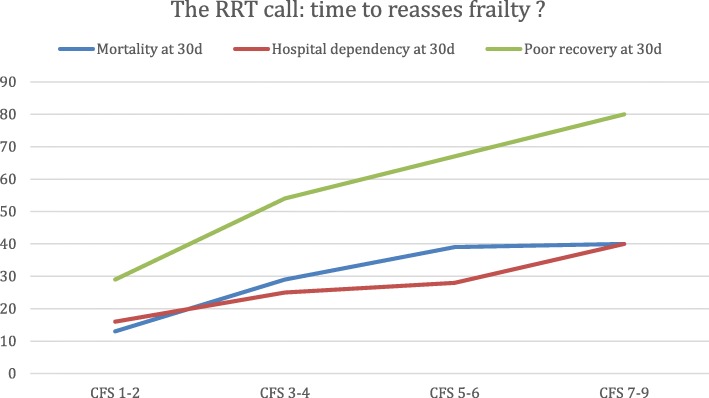
The impact of clincal frailty on 30-day outcomes is presented. The numbers on the horizontal axis indicate the ordinal values of the Dalhousie clinical frailty scale (CFS) (see “Methods”). The vertical axis indicates the percentage of patients in each category. Poor recovery is a composite measure of both mortality and hospital dependence at 30 days. RRT, rapid response team; d, days

We conducted a prospective observational study amongst 43 hospitals in 8 countries involving 1133 patients triggering RRT review, to assess the frequency and impact of clinical frailty in this patient group. Key clinical outcomes at both 24 h and 30 days are summarised in Fig. 1. We found that two fifths of patients were screened as frail - a characteristic that was associated with older age, admission under a medical specialty, increased severity of illness at the time of the RRT, and substantially, limitations of care including ICU admission. Importantly, 72% of patients who were screened as frail at the time of clinical deterioration were either dead or dependent on hospital care at 30 days. Even after adjustment for potential confounders such as age and acuity of illness, frailty remained independently associated with 30-day “poor recovery”. Figure 2 is a graphic demonstration of this result.

### Comparison with previous studies

To our knowledge, this is the first study to prospectively investigate clinical frailty in patients with significant deterioration on general wards and reviewed by the RRT. Several studies have examined the epidemiology of frailty in patients undergoing elective surgery and found it to be associated with an increased risk of post-operative adverse events [10, 11]. Similar to the findings of the present study, others have identified greater short-term mortality in patients with limitations of medical treatment at the time of an RRT call [12–14].

In this study, we were able to assess mortality and dependence on care services at 30 days. Hall and colleagues have recently also shown clinical frailty to be associated with increased 30-day mortality for patients undergoing elective surgery [15]. They used a clinical frailty score in order to assist clinicians in the planning and delivery of perioperative care for frail patients, with subsequent reductions in mortality. In patients with acute hospital admissions due to heart failure, a retrospective cohort study found the incidence of frailty to be 36% and independently associated with mortality at 30 days [16]. These data are consistent with our findings, however, our cohort captured patients from both medical and surgical specialities, with a preponderance of higher frailty scores in elderly patients admitted under medical specialities.

### Study strengths and weakness

Our study is the first to prospectively assess frailty in patients subject to RRT review. It is a prospective multi-national study, utilising standardised and previously validated data collection tools. Efforts to develop models of frailty in acute care have previously relied on large historical datasets for validation [17]. We have shown the feasibility of performing an objective frailty assessment at the time of acute care, and the biologic plausibility of the results. Refinement of this and other measures of trajectory and response to intensive care is a key research priority.

Despite our study’s strengths, many hospitals and countries contributed relatively small numbers of patients, and two thirds of data came from the UK. This potentially limits the international generalizability of our findings. Although we tested the frailty assessment tool at lead sites prior to the study commencing, we were not able to provide specific training for investigators prior to them performing patient frailty assessments at their institution; however, patient interpretation of the questions and their significance is likely to produce more variability than clinicians. Finally, a limitation of the study was the lack of data on comorbidities and admission diagnosis. Nonetheless, our results demonstrate that clinicians can make a clinical bedside assessment that is associated with patient outcome, and one that probably warrants inclusion into discussions about the goals and expectations of care. These discussions might be challenging in clinical practice, because of the lack of reliable predictors and partially because of the lack of training.

### Areas for future research

Bedside assessment of frailty may be feasible and associated with short-term outcomes, but there is a need to assess longer-term mortality and functional recovery in patients subject to RRT review and whether the high attributable mortality in these patients is at all preventable. There is also a need to better understand the interaction between frailty and clinical deterioration both in the context of acute physiology and the context of team behaviour and to explore possibilities of using frailty scores to enhance advanced care planning and end of life care.

## Conclusions

Our findings show it is feasible to assess clinical frailty in ward patients experiencing clinical deterioration subject to RRT review. Moreover, we found that frailty is associated with mortality and dependence on hospital care at 30 days amongst general ward patients.

Currently, the RRT is confronted with issues around end of life care and limiting of medical treatment in one third of all RRT calls [13] and the strong association between limitations of care and frailty suggests that there is both subjective and objective evidence that a complete recovery from illness may not be possible. Our results demonstrate that clinicians can make a clinical bedside assessment that is associated with patient outcome, and one that probably warrants inclusion into discussions about the goals and expectations of care.

We believe that using the clinical frailty scale presents an opportunity to improve advance care planning and end of life care discussions in patients subject to a RRT review. Moreover, we speculate that using the clinical frailty scale early on admission to the general ward, provided that it is implemented with dedicated and adequate training and support, one can identify (previously unrecognized) frail patients and improve the dialogue between the provider and patient and family on the expected course of recovery and/ or survivorship expectations, leading to a clear person-centered high-value treatment plan.

## Appendix

### The Canadian Study of Health and Aging Clinical Frailty Scale

1. Very fit — robust, active, energetic, well-motivated and fit; these people commonly exercise regularly and are amongst the fittest for their age.
2. Well — without active disease, but less fit than people in category 1 . Active occasionally.
3. Managing well— people whose medical problems are well controlled, but are not regularly active beyond routine walking.
4. Vulnerable — although not frankly dependent, these people commonly complain of being “slowed up” or have disease symptoms limiting activity
5. Mildly frail — more evident slowing and need help with high order ADLs (finance, transportation). Progressive impairment in shopping and walking outside alone or in doing housework.
6. Moderately frail — help is needed with all outside activities and with keeping house inside. May need help with stairs, bathing, and assistance with dressing.
7. Severely frail — completely dependent on others for personal care—either from physical or cognitive disability. May seem stable and not at high risk of dying.
8. Very severely frail — completely dependent, approaching end of life. Not likely to recover from even a minor illness
9. Terminally ill – approaching end of life. Life expectancy < 6 months who are not otherwise evidently frail.

Adapted from Rockwood et al. [5] and from Clinical Frailty Scale Version 1.2. c. 2007-2009; Geriatric Medicine Research, Dalhousie University, Halifax, NS, Canada. http://geriatricresearch.medicine.dal.ca/clinical_frailty_scale.htm. Accessed 1/12/2016.

## Abbreviations

CFS: Clinical frailty scale
CPR: Cardiopulmonary resuscitation
DNAR: Do not attempt resuscitation
MET: Medical Emergency Team
METHOD group: Medical Emergency Team Hospital Outcomes after a Day group
NEWS: National Early Warning Score
qSOFA: Quick sequential (sepsis-related) organ failure assessment
RRT: Rapid response team (includes Medical Emergency Team or Critical Care Outreach Team and denotes individuals or groups of healthcare professionals responding to deterioration hospitalised patients in locations other intensive care)
